# Optimization Design of Submerged-Entry-Nozzle Structure Using NSGA-II Genetic Algorithm in Ultra-Large Beam-Blank Continuous-Casting Molds

**DOI:** 10.3390/ma17174346

**Published:** 2024-09-02

**Authors:** Nanzhou Deng, Jintao Duan, Yibo Li, Qi Gao, Yulong Deng, Weihua Ni

**Affiliations:** 1 Light Alloy Research Institute, Central South University, Changsha 410017, China; 13277377220@163.com (N.D.);; 2 China National Heavy Machinery Research Institute Co., Ltd., Xi’an 710054, China; 3 College of Mechanical and Electrical Engineering, Central South University, Changsha 410012, China

**Keywords:** ultra-large beam blank, continuous-casting mold, submerged entry nozzle, computational fluid dynamic model, multi-objective optimization

## Abstract

To achieve uniform cooling and effective homogenization control in ultra-large beam-blank molds necessitates the optimization of submerged-entry-nozzle (SEN) structures. This study employed computational fluid dynamic (CFD) modeling to investigate the impact of two-port and three-port SEN configurations on fluid flow characteristics, free-surface velocities, temperature fields, and solidification behaviors. Subsequently, integrating numerical simulations with the non-dominated sorting genetic algorithm II (NSGA-II) and metallurgical quality-control expertise facilitated the multi-objective optimization of a three-port SEN structure suitable for beam-blank molds. The optimized parameters enabled the three-port SEN to deliver molten steel to the meniscus at an appropriate velocity while mitigating harmful effects such as SEN port backflow, excessive surface temperature differences, and shell thickness reduction due to fluid flow. The results indicated that the three-port SEN enhanced the molten-steel flow pattern and mitigated localized shell thinning compared with the two-port SEN. Additionally, the optimized design (op2) of the three-port SEN exhibited reduced boundary layer separation and superior fluid dynamics performance over the initial three-port SEN configuration.

## 1. Introduction

To meet the growing demands for high strength, toughness, and high reliability, beam blanks are evolving towards larger integral types. However, the complex cross-section of ultra-large beam blanks and the size effects of large billets lead to uneven molten-steel flow in the transition zone between the web and the flange, resulting in uneven solidification. This unevenness can result in steel breakout accidents and longitudinal cracks [1]. Additionally, the single-port straight submerged entry nozzle (SEN) commonly employed in current beam-blank continuous-casting molds often allows molten steel to penetrate deeply, thereby hindering the removal of non-metallic inclusions through flotation and preventing efficient slag-powder melting at the meniscus [2,3,4,5]. Many product defects in continuous casting correlate with molten-steel flow within the mold [6], which are significantly influenced by SEN structure, thereby affecting inclusion transport, meniscus status, solute transport, and solidification [7,8]. To ensure exceptional metallurgical quality during the casting of ultra-large beam-blank molds, a suitable new SEN type was designed.

Previous designs of beam-blank-mold SENs have primarily focused on port number, bore diameter, angle, and immersion depth. Xu et al. [9] demonstrated the impacts of different port numbers on fluid flow, heat transfer, and solidification through water modeling experiments and mathematical models. Additionally, they recommended a three-port SEN to maintain optimal meniscus conditions and consistent shell thickness at the mold exit, which reduces breakout risks from flange depressions and fillet ruptures. Furthermore, during the continuous casting of the beam-blank mold, Chen and Yang et al. [4,10,11] examined the effects of various SEN structural parameters on vortex formation, impact depth, and free-surface velocity of molten steel during continuous casting. Simulation results highlighted the bore diameter, outlet angle, immersion depth, and horizontal-port angles as crucial factors influencing molten-steel flow. Also, optimized SEN parameters according to mass calculations were recommended to enhance beam-blank cleanliness.

Several mold studies have been conducted through mathematical simulation. To elucidate fluid flow patterns inside the mold and the backflow phenomenon, as well as its controlling parameters, Garcia–Hernandez et al. [12] conducted a mathematical simulation of the fluid dynamics in the SEN and mold. Hibbeler et al. [13] developed a mathematical model for simulating the temperature profile and stress/deformation field inside the solidifying shell of a beam-blank continuous-casting mold. The model sheds light on the mechanism of crack formation in the filet region, owing to a combination of a thinner shell, excessive surface temperature differences, and mechanical stress.

Recent advancements have utilized mathematical models and optimization strategies, such as heuristic search, neural networks, genetic algorithms, and knowledge bases, which have been shown to effectively optimize product quality. Chakraborti [14,15,16] utilized genetic algorithms for optimizing continuous-casting speed through process variable adjustments. At a steel factory in Slovenia, Santos [17], developed a practical parameter-optimization approach employing a genetic algorithm and numerical simulation for the spray cooling zone during the continuous-casting process.

This study established a computational fluid dynamic (CFD) model for the solidification process of molten steel in an ultra-large beam-blank mold (900 × 510). The study investigated how two-port and three-port SEN configurations affect transient fluid flows, heat transfer, and solidification. Upon building on the research results from two SEN simulations, an interactive model integrating numerical simulations of three-port SENs with the non-dominated sorting genetic algorithm II (NSGA-II) multi-objective optimization algorithm was proposed. A knowledge base for metallurgical quality control, incorporating constraints on the process and casting quality in beam-blank molds, was connected to the algorithm. Structural constraints were also incorporated into the knowledge base to integrate design limits for the SEN. The genetic algorithm utilized the knowledge to explore spatial parameter settings and determine optimal three-port SEN structure parameters. This approach ensures compliance with SEN requirements and guarantees high productivity as well as quality in beam-blank continuous-casting molds.

## 2. Design of the SENs and CFD Models

### 2.1. Design of the SENs

**Figure 1:**
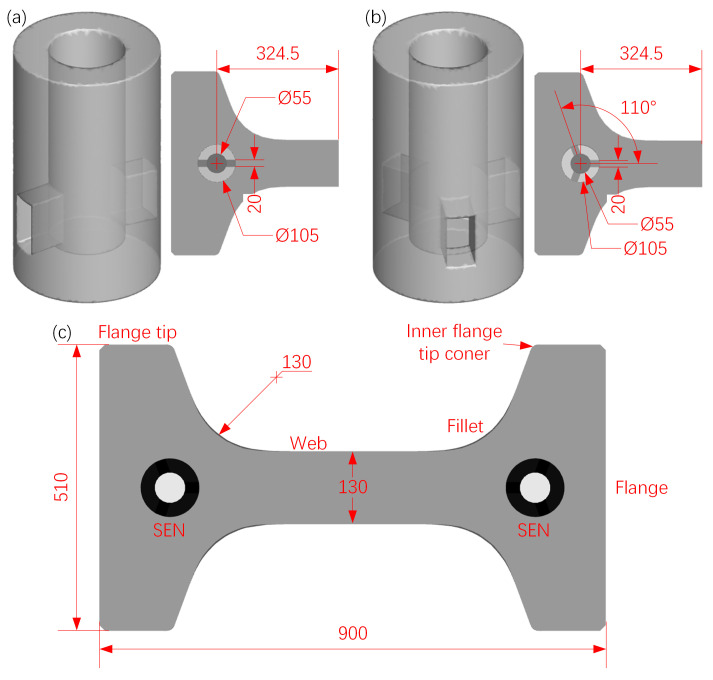
Design of (**a**) two-port SEN, (**b**) three-port SEN, and (**c**) schematic of beam-blank transverse section (mm).

This study designed two-port and three-port SENs and conducted numerical simulations to investigate their influence on metallurgical behavior in beam-blank continuous-casting molds. The outlet angle and immersion depth of these SENs were fixed at 4° and 110 mm, respectively. The geometric schematic and installation methods of SENs, as well as the schematic of the beam-blank transverse section, are shown in Figure 1.

### 2.2. Basic Assumptions of the CFD Models

Given the complexity of the fluid flow, heat transfer, and solidification of molten steel in the mold, the following presumptions were made to develop the mathematical model:

(1) The molten steel was treated as an incompressible Newton fluid.

(2) The mold’s taper and oscillation do not significantly affect fluid flow patterns.

(3) The latent heat associated with solid phase transition was negligible compared with the latent heat of solidification.

### 2.3. Governing Equations of the CFD Models

#### 2.3.1. Fluid Flow Model

Two types of fluid flow were considered within the cast strand: interdendritic flow in the mushy zone and turbulent flow in the liquid core [18]. The standard k−ε model was employed to simulate the turbulence effects of fluid flow inside the cast strand [19]. The following are expressions of the governing equations for fluid flow.

Continuity equation:

(1) $$\frac{\partial \rho }{\partial t}+\nabla \cdot \left(\rho \vec{u}\right)=0$$

where ρ is the fluid density (${\mathrm{kg}\;m}^{-3}$) and u is the velocity (${m\;s}^{-1}$).

Momentum equation:

(2) $$\frac{\partial }{\partial t}\left(\rho \vec{u}\right)+\nabla \cdot \left(\rho \vec{u}\vec{u}\right)=-\nabla P+\nabla \left[\mu_{eff}\left(\nabla \cdot \vec{u}\right)\right]+\rho \vec{g}+S_{p}$$

Turbulent kinetic energy k and turbulent dissipation rate ε equation:

(3) $$\rho \frac{\partial k}{\partial t}+\rho \left(\nabla \cdot k\vec{u}\right)=\nabla \left(\frac{\mu_{eff}}{\sigma_{k}}\cdot \nabla k\right)+G_{k}-\rho \varepsilon +S_{k}$$

(4) $$\rho \frac{\partial \varepsilon }{\partial t}+\rho \left(\nabla \cdot \varepsilon \vec{u}\right)=\nabla \left(\frac{\mu_{eff}}{\sigma_{\varepsilon }}\nabla \varepsilon \right)+C_{1}\frac{\varepsilon }{k}G_{k}-C_{2}\rho \frac{\varepsilon^{2}}{k}+S_{\varepsilon }$$

(5) $$G_{k}=\mu_{t}\frac{\partial {\vec{u}}_{i}}{\partial x_{i}}\left(\frac{\partial {\vec{u}}_{i}}{\partial x_{j}}+\frac{\partial {\vec{u}}_{j}}{\partial x_{i}}\right)$$

(6) $$\mu_{eff}=\mu +\mu_{t}$$

(7) $$\mu_{t}=\rho C_{\mu }\frac{k^{2}}{\varepsilon }$$

where P represents the static pressure (Pa); $\mu_{eff}$ is the effective viscosity, determined by summing the laminar viscosity μ and turbulent viscosity $\mu_{t}$, (${\mathrm{kg}\;m}^{-1}{\;s}^{-1}$); g denotes the gravity acceleration (${m\;s}^{-2}$); and $G_{k}$ signifies the turbulence kinetic energy resulting from the mean velocity gradient ($m^{-2}{\;s}^{-2}$). The empirical constants used in the low Reynolds-number model were $C_{1}=1.44$, $C_{2}=1.92$, $C_{\mu }=0.09$, $\sigma_{k}=1.0$, and $\sigma_{\varepsilon }=1.3$.

The mushy zone (partially solidified) was treated as a porous medium employing the enthalpy-porosity technique (EPT). The porosity of every cell was determined by the liquid percentage within the cell. Where there is total solidification, there is zero porosity, which results in the disappearance of velocity in those areas [6,20]. Additional source terms $S_{p}$, $S_{k}$ and $S_{\varepsilon }$ were added to the momentum conservation equation, turbulent kinetic energy k equation, and turbulent dissipation rate ε equation, respectively, accounting for the mushy zone’s decreased porosity:

(8) $$S_{p}=\frac{{\left(1-f_{l}\right)}^{2}}{{f_{l}}^{3}+\xi }\cdot A\cdot \left(\vec{u}-{\vec{u}}_{c}\right)$$

(9) $$S_{k}=\frac{{\left(1-f_{l}\right)}^{2}}{{f_{l}}^{3}+\xi }\cdot A\cdot k$$

(10) $$S_{\varepsilon }=\frac{{\left(1-f_{l}\right)}^{2}}{f_{l}^{3}+\xi }\cdot A\cdot \varepsilon$$

where $f_{l}$ is the liquid fraction; ξ denotes the small constant, set as 0.0001 to prevent floating point division by zero; A represents the mushy zone constant ($1\times 10^{8}$ [21]); and $u_{c}$ signifies the casting speed (${m\;s}^{-1}$).

#### 2.3.2. Heat Transfer and Solidification Model

The EPT approach was also applied to simulate the heat transfer and solidification of molten steel [22].

Energy conservation equation:

(11) $$\frac{\partial }{\partial t}\left(\rho H\right)+\nabla \left(\rho \vec{u}H\right)=\nabla \cdot \left(\lambda_{eff}\nabla T\right)+S_{h}$$

where $\lambda_{eff}$ is the effective thermal conductivity, determined by summing the thermal conductivity λ and the turbulent thermal conductivity $\lambda_{t}$, (${W\;m}^{-1}{\;K}^{-1}$), and T is the temperature (K).

The following describes the total enthalpy:

(12) $$H=h_{ref}+\int_{T_{ref}}^{T}C_{p}dT+f_{l}L$$

where $h_{ref}$ is the reference enthalpy (${J\;\mathrm{kg}}^{-1}{\;\mathrm{mol}}^{-1}$); $T_{ref}$ is the reference temperature (K); $C_{p}$ denotes the specific heat (${J\;\mathrm{kg}}^{-1}{\;K}^{-1}$); and L represents the latent heat (${J\;\mathrm{kg}}^{-1}$).

The energy conservation equation introduced a source term $S_{h}$ to explain the liquid–solid transition and the creation of the mushy zone:

(13) $$S_{h}=\rho L\left(\frac{\partial \left(1-f_{l}\right)}{\partial t}+u_{c}\nabla \left(1-f_{l}\right)\right)$$

Equation (14) was used to calculate the liquid fraction:

(14) $$f_{l}=\frac{T-T_{s}}{T_{l}-T_{s}}$$

where $T_{l}$ and $T_{s}$ are the liquidus and solidus temperatures of the molten steel (K), respectively.

### 2.4. Simulation Procedure of the CFD Models

#### 2.4.1. Simulation Models and Parameters

**Figure 2:**
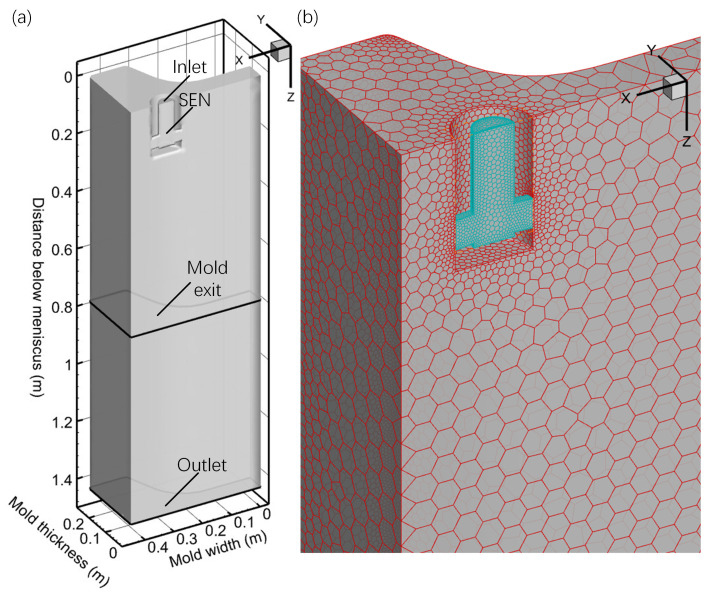
Schematic of (**a**) geometry model and (**b**) mesh structures.

The effective length of the mold was 780 mm. To prevent potential backflow at the mold exit, the computational domain was extended to 1430 mm. A polyhedral mesh structure divided the entire computational area, with local grid refinement applied to the SEN to ensure model accuracy. The domain adjacent to the mold side was enhanced with a mesh boundary layer to more accurately simulate the evolution of the solidified shell. Figure 2a depicts the geometrical model of the two-port SEN computational domain, while Figure 2b shows the mesh partition scheme for the mold. The entire mold contained ~200,000 polyhedral cells.

**Figure 3:**
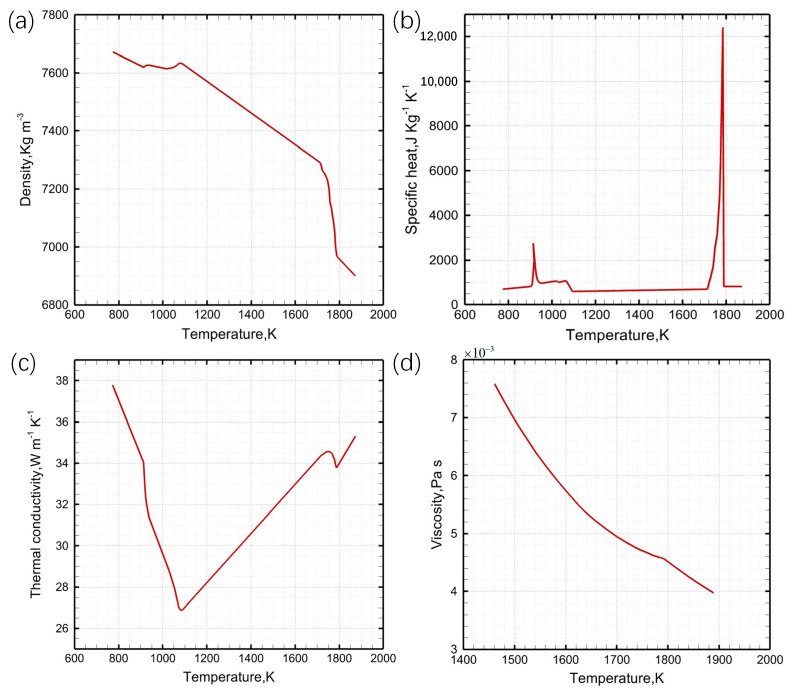
Variations of (**a**) density, (**b**) specific heat, (**c**) thermal conductivity, and (**d**) viscosity with temperature.

The physical characteristics of Q235B carbon structural steel, crucial for fluid flow, heat transfer, and solidification in the mushy zone, are presented in Table 1. Temperature-dependent variations in density, specific heat, thermal conductivity, and viscosity were calculated using the JMatpro 14.3 software (Figure 3).

#### 2.4.2. Boundary and Solution Method

Specific boundary conditions were implemented as follows: 

(1) At the SEN inlet, a velocity-inlet boundary condition was implemented. The inlet velocity was determined by the billet section and casting speed (Equation (15)).

(2) Equations (16) and (17) compute the turbulent kinetic energy (k) and turbulent dissipation rate (ε), respectively.

(3) The initial and inlet temperatures of the molten steel were determined by summing the liquidus temperature and the superheat.

(4) In the mold region, the top surface of the mold was adiabatic with zero shear stress. The heat flux across SEN walls was zero, and both mold and SEN walls were considered stationary and nonslip. The average heat flux applied to the mold walls is shown in Equation (18).

(5) The outlet assumes fully developed flow conditions, with normal gradients of all variables set to zero.

(15) $$u_{in}=\frac{u_{cast}\cdot S_{out}}{S_{SEN}}$$

(16) $$k=0.01u_{in}^{2}$$

(17) $$\varepsilon =\frac{k^{1.5}}{D_{SEN}}$$

(18) $$\bar{q}=\frac{\rho_{w}C_{p}Q_{w}\Delta T_{water}}{S_{eff}}$$

where $S_{out}$ and $S_{SEN}$ are the areas of the mold outlet and SEN inlet ($m^{2}$), respectively; $D_{SEN}$ is the hydraulic diameter of the SEN (m); $\rho_{w}$ is the cooling water density (${\mathrm{kg}\;m}^{-3}$); $C_{p}$ is the specific heat of the cooling water (${J\;\mathrm{kg}}^{-1}{\;K}^{-1}$); $Q_{w}$ is the cooling water flow ($m^{3}{\;s}^{-1}$); and $\Delta T_{water}$ is the temperature difference in the cooling water between the inlet and outlet of the copper tube (K). $S_{eff}$ represents the effective contact area between the molten steel and mold walls ($m^{2}$).

The flow and heat transfer calculations incorporate the casting speed, $u_{cast}=0.75\;{m\;s}^{-1}$, superheat of molten steel, $T_{superheat}=25\;K$, cooling water flow, $Q_{w}{=8800\;L\;\min}^{-1}$, and temperature difference between the inlet and outlet of the copper tube, $\Delta T_{water}=4\;K$.

Transient calculations were performed using the ANSYS Fluent software, and a pressure-based solver was employed to solve the governing equations. The algorithm for pressure–velocity coupling is known as SIMPLEC. The discretization scheme for the pressure algorithm was PRESTO, while a second-order upwind discretization scheme was applied for momentum. Convergence criteria were met when the energy residual was $\leq 10^{-6}$, and other residuals were $\leq 10^{-4}$.

## 3. Comparison of Fluid Flow, Heat Transfer, and Solidification for Two-Port SEN and Three-Port SEN

### 3.1. Fluid Flow

**Figure 4:**
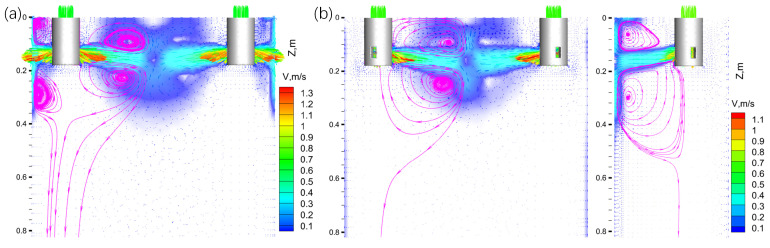
Comparison of velocity vectors for (**a**) two-port SEN and (**b**) three-port SEN.

To visually illustrate the flow patterns in the mold for two-port and three-port SENs, Figure 4 depicts the velocity vectors on the x–z plane (y = 0 m) and the plane formed by x–z at 70°. Radial-flow SENs exhibited a “self-braking” effect that enhanced flow stability in the web region [9]. Molten steel from two SEN ports was directed toward the web center. Upon collision, a component of the flow increased spirally to the surface while the other half spirally descended, thereby creating the well-known “double roll” pattern [23]. The upward stream contributed to an active meniscus with increased temperature.

Figure 4a, depicts the flow pattern of the two-port SEN near the flange. Upon impact with the initially solidified shell of the flange at a high velocity of 1.3 m/s, the SEN jet flow divided into two longitudinal circulations. A strong upper circulation centered at 0.04 m below the meniscus close to the free surface induced significant surface fluctuations. The circular flow below the SEN, centered at 0.3 m below the meniscus and close to the flange, did not facilitate the effective removal of non-metallic inclusions near the mold center [24]. Contrastingly, Figure 4b illustrates the injection of molten steel at lower velocities toward the mold corners with the three-port SEN, owing to the longer distance between the nozzle outlets and mold corners. The centers of upper and lower circulations, located at 0.06 m and 0.3 m below the meniscus, respectively, exhibited significantly weaker upper circulations and a shift in the impact zones of circular flow toward the mold center, thereby facilitating meniscus stability and inclusion removal.

**Figure 5:**
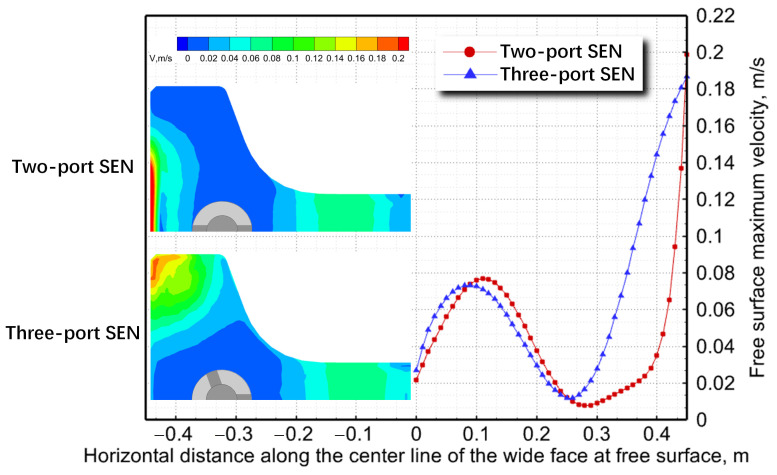
Comparison of maximum free-surface velocity for two-port SEN and three-port SEN.

Figure 5 shows the predicted maximum velocity on the free surface, which was obtained 20 mm below the meniscus, for both SEN types. The molten-steel flow conditions below the meniscus significantly influenced free-surface velocity. The two-port SEN exhibited a notably higher rate of change in free-surface velocity near the flange compared with the three-port SEN. A maximum free-surface velocity of 0.2 m/s at the flange center increased the risk of steel/slag interface damage and slag entrainment [25]. Additionally, the velocity distribution on the free surface of the three-port SEN was more uniform compared with the two-port SEN, thereby effectively preventing the formation of a dead zone and the freezing of molten steel [26]. Thus, comprehensively considering flow characteristics in both models, the three-port SEN exhibited superior metallurgical effects.

### 3.2. Temperature Field and Solidification

**Figure 6:**
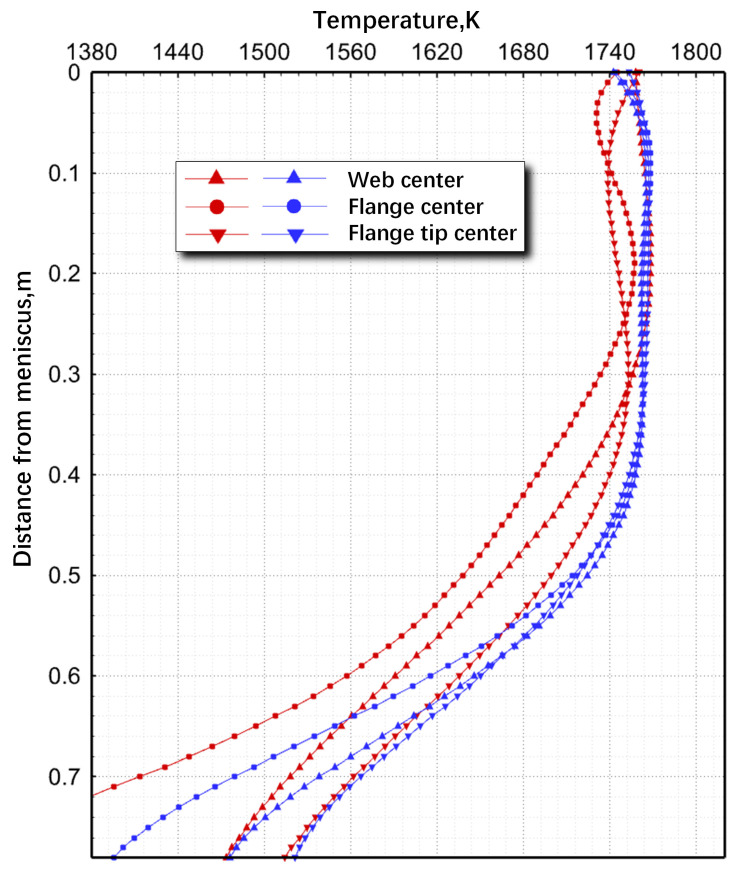
Comparison of surface temperature variation along the casting direction for two-port SEN (red lines) and three-port SEN (blue lines).

Figure 6 illustrates the temperature distribution along the casting direction with two distinct types of SEN. As the molten-steel flow enters the impacting zone, the surface temperature at the center of the two-port SEN flange increased from 1731 K at 45 mm to 1757 K at 192 mm below the meniscus. Upon exiting this zone, the surface temperature decreased. The temperature variation observed with the three-port SEN exhibited similar trends compared with the two-port SEN. Both exhibited localized high-temperature zones. These findings indicated the close relationship between temperature distribution and the flow pattern of molten steel within the mold. Despite variations in different SEN configurations, temperature changes in the impact zone remained consistent. The direct impingement of high-temperature molten steel from SEN ports on the solidification front resulted in localized maximum superheat, with heat dissipating through longitudinal circulation. Notably, the circumferential temperature distribution of the three-port SEN remained more uniform across the web center, flange center, and flange tip center, thereby reducing the possibility of cracks and defects [1].

**Figure 7:**
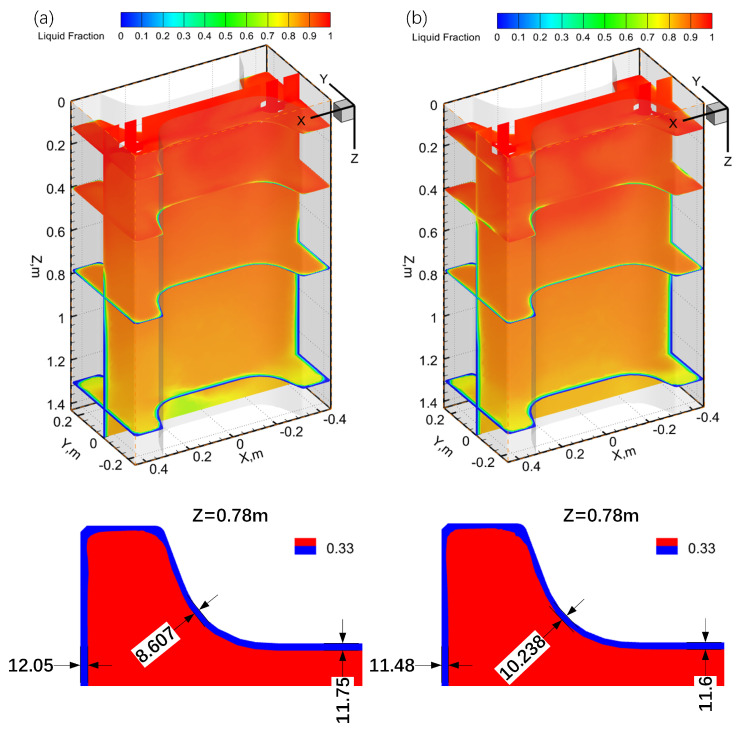
Comparison of the liquid fraction at various cross-sections for (**a**) two-port SEN and (**b**) three-port SEN.

Figure 7 shows the liquid fraction at different cross-sections under both types of SEN. In this study, a liquid fraction of 0.33 defined the solidification front. Under the two-port SEN, the thickness of the solidified shell at the mold-outlet (z = 0.78 m) web center, flange center, and fillet measured 11.75 mm, 12.05 mm, and 8.607 mm, respectively. For the three-port SEN, these values were 11.6 mm, 11.48 mm, and 10.238 mm, indicating a maximum thickness change reduction of 2.081 mm compared with the two-port SEN. This difference was attributable to the fact that ports of the three-port SEN were farther from the mold wall, thereby resulting in lower molten-steel impact velocities compared with the two-port SEN. Consequently, the homogeneity of the solidified shell was less affected by shock pressure from jet flows against the mold wall. Thus, the solidification behavior of the three-port SEN was more acceptable within the mold, potentially reducing the risk of liquid breakout [25].

## 4. Multi-Objective Optimization Model for Three-Port SEN Structure

The multi-objective optimization model integrates the NSGA-II algorithm with CFD models. The NSGA-II algorithm, which encompasses optimization strategies and a set of constraints, was employed to determine the optimization parameters for the SEN structure and to integrate new input variables into the CFD model. The CFD model simulates the flow, heat transfer, and solidification behavior, with the results being fed back to the NSGA-II algorithm. Given the metallurgical phenomena of these two types of SEN (Section 3), the three-port SEN was selected as the focus of optimization for this study.

### 4.1. Knowledge Base Representation for Metallurgical Quality Control

The criteria below are structured in terms of the flow and thermal behavior of molten steel in the mold, thereby drawing cause-and-effect relationships from the literature.

(1) Shell thickness at the mold exit ($S_{m}$) [27].

Hot molten steel with high momentum impinging against the solidified shell resulted in the thinning of the shell, thereby increasing the risk of deformation and breakout under pressure. It was crucial to maintain a shell thickness at the mold exit above a specific minimum value, preventing breakout due to liquid ferrostatic pressure. The minimum acceptable value was ~12 mm.

$$S_{m}\geq 12$$

(2) The velocity of the free surface ($V_{s}$) [28,29].

An optimal velocity range for molten steel at the free surface was crucial as too low a velocity may result in freezing near the meniscus, impeding proper melting of mold flux. However, an excessively high value can lead to excessive level fluctuation and slag entrainment. An acceptable free-surface velocity range was 0.1–0.4 m/s.

$$0.1\leq V_{s}\leq 0.4$$

(3) The effective area fraction at the port (β) [12,30,31].

The effective area fraction represents the flow fraction with a positive directional velocity component at the port area, ensuring it exits the SEN domain effectively. Maximally, the design of the SEN minimized or eliminated backflow at each port area, enhancing the utilization of the effective outlet area, thereby reducing jet velocity and achieving a more stable flow pattern.

(4) The temperature difference between the fillet and inner-flange tip corner at the mold exit (ΔT) [32,33].

In continuous-casting mold for beam blanks, the fillet and inner-flange tip corners exhibited the highest and lowest wide surface temperatures, respectively. The temperature difference between them served as an indicator of hot surface temperature uniformity. To prevent, longitudinal cracks at the fillet, the SEN design should minimize the temperature difference at the mold exit.

### 4.2. Definition of the Optimization Issues

Multi-objective optimization design was conducted on the primary parameters of the three-port SEN, guided by relevant performance indicators from the metallurgical quality-control knowledge base. The aim was to obtain a superior SEN structure, enhanced flow, and heat transfer performance. The objective includes maximizing the effective area fraction at the port and minimizing the temperature difference between the fillet and the inner-flange tip corner. Additionally, constraints were placed on the shell thickness at the mold exit and the velocity of the free surface to prevent significant damage. The design variables include the bore diameter ($t_{1}$), the outlet angle ($t_{2}$), the immersion depth ($t_{3}$), and the port’s horizontal angles ($t_{4}$) of the three-port SEN. They ranged from 45 mm to 50 mm for $t_{1}$, from 2° to 15° for $t_{2}$, from 110 mm to 126 mm for $t_{3}$, and from 104° to 124° for $t_{4}$, respectively. The size of the square port was calculated according to the aspect ratio of 2:1 and equal-area principles relative to the SEN inlet. The optimization issue can be expressed mathematically as follows:

(19) $$\left\{\begin{array}{ll} Min & \left[\Delta T(X),-\beta (X)\right]\\ s.t. & X=(t_{1},t_{2},t_{3},t_{4})\\ & S_{m}(X)\geq 12\\ & 0.1\leq V_{s}(X)\leq 0.4\\ & 45\leq t_{1}\leq 50\\ & 2\leq t_{2}\leq 15\\ & 110\leq t_{3}\leq 126\\ & 104\leq t_{4}\leq 124 \end{array}\right.$$

### 4.3. Surrogate Models and Error Metrics

Generally, formulating objective functions for complex nonlinear issues can be challenging. As an effective alternative, the polynomial response surface method (PRSM) was employed to predict response values for unknown points according to the information from known points. This approach facilitated design optimization with relatively low computational cost [34]. The PRSM model utilizes a quartic polynomial function in subsequent optimization procedures, defined as

(20) $$\begin{array}{cc} F(X) & =a_{0}+a_{1}t_{1}+a_{2}t_{2}+a_{3}t_{3}+a_{4}t_{4}+a_{5}t_{1}^{2}+a_{6}t_{2}^{2}+a_{7}t_{3}^{2}+a_{8}t_{4}^{2}+a_{9}t_{1}t_{2}+a_{10}t_{1}t_{3}+a_{11}t_{1}t_{4}\\ & +a_{12}t_{2}t_{3}+a_{13}t_{2}t_{4}+a_{14}t_{3}t_{4}+a_{15}t_{1}^{3}+a_{16}t_{2}^{3}+a_{17}t_{3}^{3}+a_{18}t_{4}^{3}+a_{19}t_{1}^{4}+a_{20}t_{2}^{4}+a_{21}t_{3}^{4}+a_{22}t_{4}^{4} \end{array}$$

Design of experiments (DOE), including full factorial design, Latin hypercube, central composite design, and Taguchi orthogonal array, were employed to generate sampling points for determining PRSM coefficients [35]. To construct a high-precision surrogate model, 30 training points were selected in the design space using Latin hypercube sampling. Furthermore, to evaluate the accuracy of the model, five additional validation points distributed over the design domain were randomly generated. The accuracy of the surrogate model was validated using R-square ($R^{2}$), root-mean-square error (RMSE), maximum absolute percentage error (MAPE), and relative error (RE) (Equations (21)–(24)) [36].

(21) $$R^{2}=1-\frac{\sum_{i=1}^{n}{\left(y_{i}-{\hat{y}}_{i}\right)}^{2}}{\sum_{i=1}^{n}{\left(y_{i}-{\bar{y}}_{i}\right)}^{2}}$$

(22) $$RMSE=\sqrt{\frac{1}{n}\sum_{i=1}^{n}{\left({\hat{y}}_{i}-y_{i}\right)}^{2}}$$

(23) $$MAPE=\max\left(\left|\frac{{\hat{y}}_{i}-y_{i}}{y_{i}}\right|\right)\times 100\%$$

(24) $$RE=\frac{y_{i}-{\hat{y}}_{i}}{{\hat{y}}_{i}}\times 100\%$$

where n denotes the number of sample points; $y_{i}$ and $\hat{y}$ are the exact function value of the ith sample point and the corresponding value of the surrogate model; and $\bar{y}$ represents the average value of $y_{i}$.

The values of these performance metrics revealed the predictive accuracy and capability of the surrogate models at new points. The overall accuracy of the approximate model was estimated using $R^{2}$ and RSME, while the local accuracy was calculated using MAPE. Generally, the surrogate model is deemed more accurate when $R^{2}$ approaches unity, and RMSE and RAPE values are smaller.

### 4.4. Multi-Objective Optimization Algorithm and Procedure

**Figure 8:**
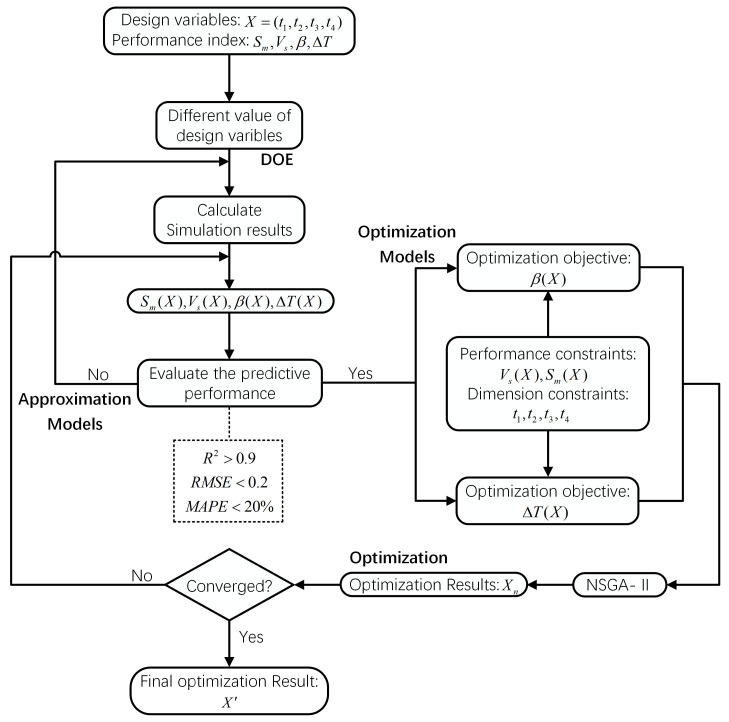
Overall optimization process.

The NSGA-II algorithm, known for its efficiency in multi-objective optimization through non-dominated sorting, was adopted [34,37]. In this study, NSGA-II was employed to obtain Pareto fronts of two objectives: ΔT and β. Details of NSGA-II parameter definitions are summarized in Table 2. Furthermore, Figure 8 shows a flowchart illustrating the multi-objective optimization process for the three-port SEN structure. 

## 5. Results and Discussion of Three-Port SEN Structure Optimization

### 5.1. Error Analysis of Surrogate Models

In this section, the relationship between design variables and performance indicators was established using the DOE and PRSM. Each set of training points was inputted into the Fluent software to calculate their flow and heat transfer processes. Subsequently, multi-objective optimization was conducted (Figure 8) after each simulation. Ultimately, 28 out of the 30 sets of sampling points were reserved as effective data to establish the approximate model for each performance indicator. Equations (25)–(28) derive quartic polynomial functions for $S_{m}$, $V_{s}$, β, and ΔT versus the three-port SEN structural parameters $x_{1}$, $x_{2}$, $x_{3}$, and $x_{4}$. Among $x_{1}$, $x_{2}$, $x_{3}$, and $x_{4}$ were the normalization processing results of $t_{1}$, $t_{2}$, $t_{3}$, and $t_{4}$ (Equation (29)).

**Figure 9:**
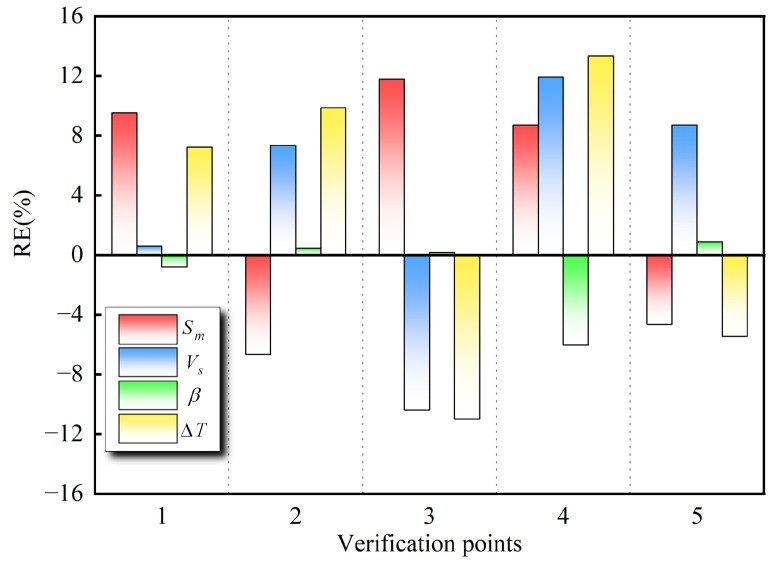
Relative error of the CFD results and surrogate models at five verification points.

Table 3 presents the modeling accuracies of the PRSM functions established according to the simulation results of the Latin hypercube design, thereby explicitly comparing the performance of these surrogate models. It was evident that the $R^{2}$ value was ~1, and the values of RAAE and MAPE were also within 0.2. Additionally, the RE errors of the CFD results and surrogate models at the five verification points were all less than 0.2 (Figure 9). In this study, despite the quartic polynomial not achieving optimal modeling accuracy, it met the requirements for subsequent analysis.

(25) $$\begin{array}{cc} S_{m}(x_{1},x_{2},x_{3},x_{4}) & =-0.739545-11.616679x_{1}-15.262128x_{2}+7.723633x_{3}+66.054920x_{4}+69.60035x_{1}^{2}+125.318388x_{2}^{2}\\ & +15.110956x_{3}^{2}-181.559462x_{4}^{2}-5.495696x_{1}\cdot x_{2}-1.942153x_{1}\cdot x_{3}-6.978725x_{1}\cdot x_{4}-9.503054x_{2}\cdot x_{3}\\ & -22.805053x_{2}\cdot x_{4}-9.232982x_{3}\cdot x_{4}-118.949797x_{1}^{3}-161.693642x_{2}^{3}-29.785325x_{3}^{3}+251.970699x_{4}^{3}\\ & +64.453376x_{1}^{4}+65.847090x_{2}^{4}+16.172380x_{3}^{4}-120.212116x_{4}^{4} \end{array}$$

(26) $$\begin{array}{cc} V_{s}(x_{1},x_{2},x_{3},x_{4}) & =0.799205+0.385406x_{1}-0.196457x_{2}-0.395435x_{3}-2.263223x_{4}-3.600791x_{1}^{2}-3.292458x_{2}^{2}\\ & -0.088688x_{3}^{2}+6.155039x_{4}^{2}+0.174842x_{1}\cdot x_{2}+0.260654x_{1}\cdot x_{3}+0.582022x_{1}\cdot x_{4}+0.474829x_{2}\cdot x_{3}\\ & +0.840833x_{2}\cdot x_{4}+0.244096x_{3}\cdot x_{4}+4.988544x_{1}^{3}+5.713356x_{2}^{3}-0.487384x_{3}^{3}-9.180052x_{4}^{3}\\ & -2.257325x_{1}^{4}-2.970844x_{2}^{4}+0.570349x_{3}^{4}+4.689092x_{4}^{4} \end{array}$$

(27) $$\begin{array}{cc} \beta (x_{1},x_{2},x_{3},x_{4}) & =0.932077+0.496093x_{1}+0.201764x_{2}-0.066214x_{3}-0.885431x_{4}-2.896515x_{1}^{2}-1.673432x_{2}^{2}\\ & +0.055477x_{3}^{2}+3.360726x_{4}^{2}+0.171254x_{1}\cdot x_{2}+0.024799x_{1}\cdot x_{3}+0.098588x_{1}\cdot x_{4}+0.046374x_{2}\cdot x_{3}\\ & +0.259539x_{2}\cdot x_{4}-0.016215x_{3}\cdot x_{4}+4.734754x_{1}^{3}+2.406439x_{2}^{3}+0.212107x_{3}^{3}-4.976278x_{4}^{3}\\ & -2.448573x_{1}^{4}-1.038130x_{2}^{4}-0.260762x_{3}^{4}+2.407250x_{4}^{4} \end{array}$$

(28) $$\begin{array}{cc} \Delta T(x_{1},x_{2},x_{3},x_{4}) & =45.523472+527.365078x_{1}+828.070365x_{2}-32.616692x_{3}+389.624848x_{4}-5224.898047x_{1}^{2}\\ & -2426.515251x_{2}^{2}+2187.626392x_{3}^{2}-722.916239x_{4}^{2}+420.612841x_{1}\cdot x_{2}+64.653624x_{1}\cdot x_{3}\\ & +184.190949x_{1}\cdot x_{4}-206.869589x_{2}\cdot x_{3}-180.269063x_{2}\cdot x_{4}-196.171962x_{3}\cdot x_{4}+10203.228028x_{1}^{3}\\ & +2421.183173x_{2}^{3}-4411.343522x_{3}^{3}+391.519852x_{4}^{3}-5803.911891x_{1}^{4}-499.073543x_{2}^{4}\\ & +2461.750777x_{3}^{4}+89.707704x_{4}^{4} \end{array}$$

(29) $$x_{1}=\frac{t_{1}-45}{5},x_{2}=\frac{t_{2}-2}{13},x_{3}=\frac{t_{3}-110}{16},x_{4}=\frac{t_{4}-104}{20}$$

### 5.2. Multi-Objective Optimization Results

**Figure 10:**
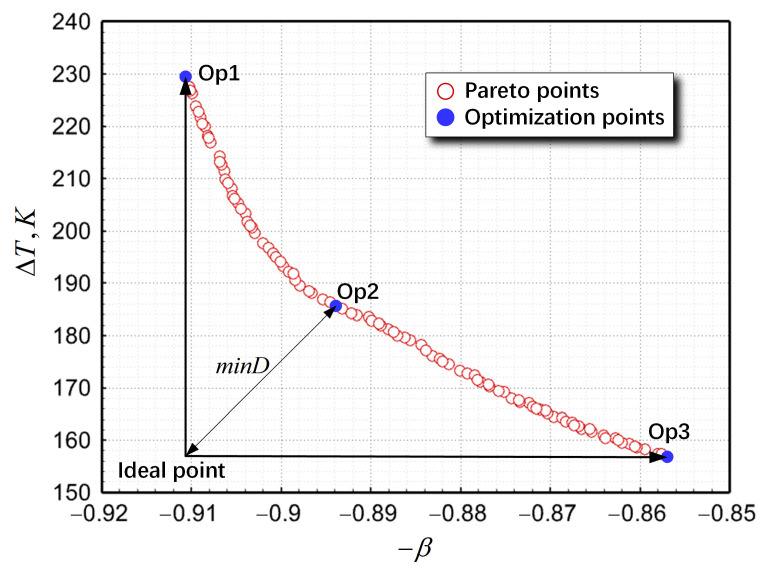
Pareto solutions from the NSGA-II algorithm.

Figure 10 illustrates the Pareto fronts for optimizing the three-port SEN structure using the NSGA-II algorithm. Notably, two goals—β and ΔT—contradicted one another, as an increase in β invariably increased ΔT. Therefore, the optimal design requires balancing practical engineering needs. Upon focusing solely on β, the optimization of point 1 (op1)’s design features a bore diameter of 45 mm, an outlet angle of 11°, an immersion depth of 110 mm, and port horizontal angles of 114°. The actual value β, derived using the CFD model, was 0.9162, and was slightly higher compared with the surrogate model prediction of 0.9106. Conversely, given the single objective ΔT, the op3 design was obtained with a bore diameter of 46 mm, an outlet angle of 9°, an immersion depth of 111 mm, and port horizontal angles of 112°. In this design, the values of β and ΔT were 0.8570 and 156.9 K, respectively.

To balance β and ΔT, a compromise solution of op2 design was obtained based on the “distance minimization” criterion (Equation (30) [35]).

(30) $$minD={\left\{{\sum_{i=1}^{u}\left[F_{i}^{k}-min\left(F_{i}\right)\right]}^{2}\right\}}^{1/2}$$

where u is the number of objective functions, u=2, and $F_{i}^{k}$ is the objective function i at the optimization point k.

The optimized SEN featured a bore diameter of 45 mm, an outlet angle of 10°, an immersion depth of 110 mm, and port horizontal angles of 114°. In comparison with the initial three-port SEN, β increased by 9.20% and ΔT decreased by 45.12%, and $S_{m}$ and $V_{s}$, met metallurgical standards. Additionally, Table 4 presents the surrogate model predictions with the CFD model results to validate their accuracy. It was observed that the maximum relative error of β was <4.26% and the error of ΔT was <12.45% between CFD results and surrogate models. This further indicated that optimization results based on the PRSM model achieved a high level of accuracy.

### 5.3. Discussion of the Three-Port SEN Structure Optimization

**Figure 11:**
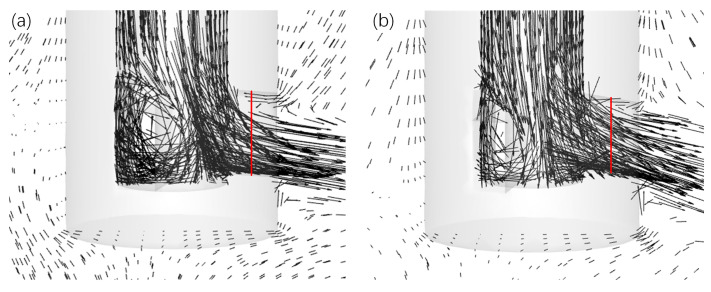
Velocity profiles at the central symmetrical plane of (**a**) initial three-port SEN and (**b**) op2 design.

Figure 11 illustrates the velocity profiles at the outlet port of the initial three-port SEN and the op2 design, examining the recirculation effect. It was observed that fluid recirculated backward at the upper edge of the port, thereby indicating an incomplete utilization of the entire outlet area.

**Figure 12:**
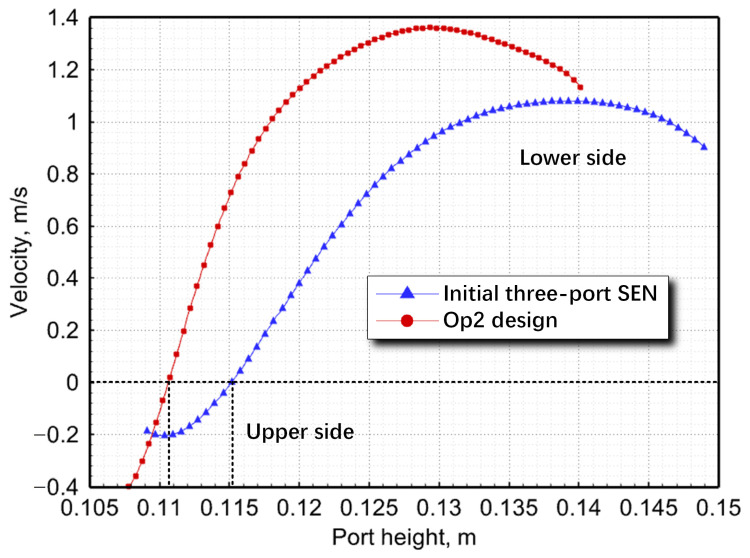
Velocity magnitude along a line from upper to lower sides of the SEM port center.

To quantify the backflow phenomenon, a line (Figure 11) parallel to the SEN axis was drawn through the center of the port to analyze velocity magnitudes (Figure 12). Overall, as molten steel rebounded after impinging at the bottom of the SEN, the velocity gradually decreased from the lower to upper sides, eventually leading to backflow. The effective area fraction of SEN increased by 9.20% post-optimization, indicating enhanced steel-flow delivery with reduced backflow compared with the initial design. Given this phenomenon, it was observed that jets exhibited a degree of dynamic distortion, thereby necessitating the further fluid dynamic analysis of the SEN [12,26].

**Figure 13:**
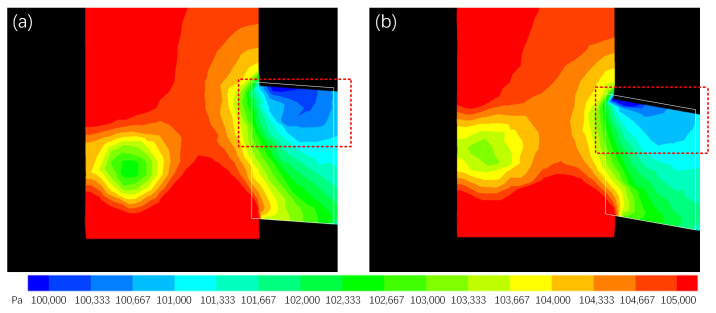
Pressure contours at the center symmetrical plane of (**a**) initial three-port SEN and (**b**) op2 design.

**Figure 14:**
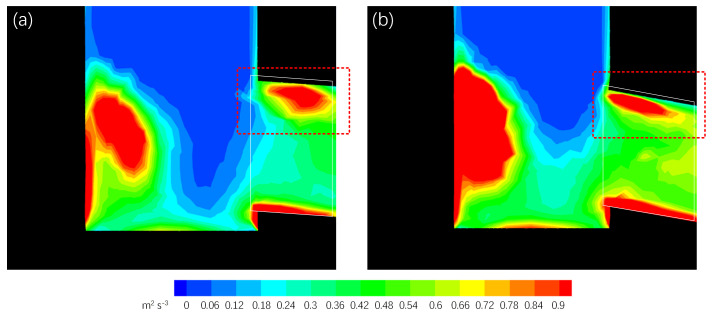
Kinetic energy dissipation contours at the center symmetrical plane of (**a**) the initial three-port SEN and (**b**) op2 design.

Given these findings and principles from boundary layer theory, it was observed that boundary layer separation occurred when molten steel reached the upper internal side of the port. This separation created low-pressure zones with significant velocity changes, resulting in high kinetic energy dissipation (Figure 13 and Figure 14). The initial three-port SEN exhibited spatial inhomogeneity with a large low-pressure zone at the upper side of the port (Figure 13). Conversely, the op2 design exhibited fewer low-pressure zones in the backflow area. Additionally, the exiting flow of the optimized SEN was homogeneously distributed with reduced energy dissipation (Figure 14).

The op2 design effectively mitigated backflow at the port outlet, optimizing the use of the entire outlet area. It exhibited superior fluid dynamics performance, ensuring more uniform and stable molten-steel flow in the mold, thereby enhancing the continuous-casting processes. Thus, optimizing the SEN structure through a multi-objective approach proved beneficial for enhancing performance.

## 6. Conclusions

Upon conducting numerical simulations and multi-objective optimization modeling, this study investigated the influence of different SENs on fluid flow, heat transfer, and solidification in an ultra-large beam-blank continuous-casting mold. A new SEN structure design was developed based on these findings. The analysis of simulation results led to the following conclusions:

(1) The maximum jet velocity of the three-port SEN was reduced to 1.1 m/s, while the two-port SEN was 1.3 m/s. Additionally, owing to the longer distance between the three-port SEN and the mold wall, the change rate in free-surface velocity was significantly smaller compared with the two-port SEN, and the velocity distribution was more uniform.

(2) The three-port SEN ensured a more consistent mold-outlet thickness distribution compared with the two-port SEN and exhibited a more acceptable surface temperature trend along the casting direction. This design reduces the risk of breakout due to skin rupture at the fillet.

(3) The op2 design of the three-port SEN demonstrated an enhanced effective area fraction at the port compared with the pre-optimized SEN, thereby reducing boundary layer separation and mitigating low-pressure areas and high dissipation to a considerable extent. Furthermore, the optimized SEN exhibited enhanced fluid dynamics and achieved a more stable flow pattern.

This study underscored the effectiveness of the new SEN design in enhancing casting quality and operational stability in ultra-large beam-blank continuous-casting processes. Please note that surrogate models are not limited to PRSM; exploring alternative approaches is recommended. For more information on methods that can be employed, please refer to citation [38].

## Figures and Tables

**Table 1:** Physical parameters of the Q235B.

Parameters	Values
Latent heat of steel [${J\;\mathrm{Kg}}^{-1}$]	274,129
Solidus of steel [K]	1715.65
Liquidus of steel [K]	1785.47

**Table 2:** Parameters for the NSGA-II algorithm.

Parameters	Values
Population size	100
Number of function evolutions	500
Crossover probability	0.7
Mutation probability	0.4
Initialization mode	Random

**Table 3:** Accuracies of the PRSM surrogate models.

Objective	$S_{m}$	$V_{s}$	β	ΔT
$R^{2}$	0.9215	0.9452	0.9502	0.9728
RMSE	0.031	0.076	0.052	0.049
MAPE (%)	7.75	12.56	1.64	9.26

**Table 4:** Comparison of optimal and initial designs for the three-port SEN.

Objective	Initial	Op1 Design			Op2 Design			Op3 Design		
$t_{1}$ (mm)	55	45			45			46		
$t_{2}$ (°)	4	11			10			9		
$t_{3}$ (mm)	110	110			110			111		
$t_{4}$ (°)	110	114			114			112		
	CFD	CFD	PRSM	RE (%)	CFD	PRSM	RE (%)	CFD	PRSM	RE (%)
$S_{m}$ (mm)	9.26	12.47	12.24	1.87	12.08	12.14	−0.49	11.09	11.71	−5.29
$V_{s}$ (m/s)	0.1840	0.1999	0.1957	2.14	0.1972	0.1985	−0.65	0.1808	0.1649	9.64
β	0.8535	0.9162	0.9106	0.61	0.9321	0.8940	4.26	0.8931	0.8570	4.21
Increase		7.34%			9.20%			4.63%		
ΔT (K)	328.00	258.00	229.42	12.45	180.00	185.43	−2.92	176.00	156.92	12.15
Increase		−21.34%			−45.12%			−46.34%		

## Data Availability

Data are contained within the article.

## References

[1] Lee J.-E., Yeo T.-J., Hwan OH K., Yoon J.-K., Yoon U.-S. (2000). Prediction of cracks in continuously cast steel beam blank through fully coupled analysis of fluid flow, heat transfer, and deformation behavior of a solidifying shell. Metall. Mater. Trans. A.

[2] Peixoto J.J.M., Gabriel W.V., Ribeiro L.Q., Silva C.A.d., Silva I.A.d., Seshadri V. (2016). Computational and physical simulation of fluid flow inside a beam blank continuous casting mold. J. Mater. Process. Technol..

[3] Chen W., Zhang Y., Zhu L., Zhang C., Chen Y., Wang B., Wang C. (2012). Three-dimensional FEM study of fluid flow in mould for beam blank continuous casting: Influence of straight through conduit type SEN. Ironmak. Steelmak..

[4] Chen W., Zhang Y., Zhu L., Zhang C., Chen Y., Wang B., Wang C. (2012). Three-dimensional FEM study of fluid flow in mould for beam blank continuous casting: Influence of nozzle structure and parameters on fluid flow. Ironmak. Steelmak..

[5] Huang Z., Yang X., Gao Q., Chen Z., Long M., Chen D. (2024). The influence of SEN structure on the flow and solidification phenomena in ultra-large-section beam blank mould. J. Mater. Res. Technol..

[6] Li X., Zhang Z., Fang M., Liu K. (2022). Numerical Simulation of the Fluid Flow, Heat Transfer, and Solidification in Ultrahigh Speed Continuous Casting Billet Mold. Steel Res. Int..

[7] Thomas B.G., Zhang L. (2001). Mathematical modeling of fluid flow in continuous casting. ISIJ Int..

[8] Xu P., Zhou Y.-z., Chen D.-f., Long M.-j., Duan H.-m. (2022). Optimization of submerged entry nozzle parameters for ultra-high casting speed continuous casting mold of billet. J. Iron Steel Res. Int..

[9] Xu M., Zhu M. (2015). Transport phenomena in a beam-blank continuous casting mold with two types of submerged entry nozzle. ISIJ Int..

[10] Yang J.-w., Du Y.-p., Shi R., Cui X.-C., Liu C. (2004). Effect of SEN parameters on 3D flow field in mould of beam blank continuous caster. J. Iron Steel Res. Int..

[11] Zhang L., Chen D., Long M., Xie X., Zhang X., Ma Y. Hydraulic simulation of fluid flow in beam blank continuous casting mold with double nozzles. Proceedings of the EPD Congress 2014.

[12] Garcia-Hernandez S., Morales R.D., Barreto J.d.J., Morales-Higa K. (2013). Numerical Optimization of Nozzle Ports to Improve the Fluidynamics by Controlling Backflow in a Continuous Casting Slab Mold. ISIJ Int..

[13] Hibbeler L.C., Xu K., Thomas B.G., Koric S., Spangler C. (2009). Thermomechanical modeling of beam blank casting. Iron Steel Technol..

[14] Chakraborti N., Kumar K.S., Roy G. (2003). A heat transfer study of the continuous caster mold using a finite volume approach coupled with genetic algorithms. J. Mater. Eng. Perform..

[15] Chakraborti N., Kumar R., Jain D. (2001). A study of the continuous casting mold using a pareto-converging genetic algorithm. Appl. Math. Model..

[16] Chakraborti N., Mukherjee A. (2000). Optimisation of continuous casting mould parameters using genetic algorithms and other allied techniques. Ironmak. Steelmak..

[17] Santos C.A., Cheung N., Garcia A., Spim J.A. (2005). Application of a Solidification Mathematical Model and a Genetic Algorithm in the Optimization of Strand Thermal Profile Along the Continuous Casting of Steel. Mater. Manuf. Process..

[18] Yang H., Zhang X., Deng K., Li W., Gan Y., Zhao L. (1998). Mathematical simulation on coupled flow, heat, and solute transport in slab continuous casting process. Metall. Mater. Trans. B.

[19] Jones W.P., Launder B. (1972). The prediction of laminarization with a two-equation model of turbulence. Int. J. Heat Mass Transf..

[20] Chen W., Ren Y., Zhang L. (2018). Large eddy simulation on the fluid flow, solidification and entrapment of inclusions in the steel along the full continuous casting slab strand. JOM.

[21] Trindade L., Nadalon E., Contini A., Barroso R. (2016). Modeling of Solidification in Continuous Casting Round Billet with Mold Electromagnetic Stirring (M-EMS). Steel Res. Int..

[22] Brent A.D., Voller V.R., Reid K.J. (1988). Enthalpy-Porosity Technique for Modeling Convection-Diffusion Phase Change: Application to the Melting of a Pure Metal. Numer. Heat Transf..

[23] Huang C., Zhou H., Zhang L., Yang W., Zhang J., Ren Y., Chen W. (2021). Effect of Casting Parameters on the Flow Pattern in a Steel Continuous Casting Slab Mold: Numerical Simulation and Industrial Trials. Steel Res. Int..

[24] Fang Q., Ni H., Zhang H., Wang B., Song X., Liu C. (2018). Influence of SEN on flow, solidification, and solute transport in bloom casting mold. JOM.

[25] Fang Q., Ni H., Zhang H., Wang B., Lv Z. (2017). The Effects of a Submerged Entry Nozzle on Flow and Initial Solidification in a Continuous Casting Bloom Mold with Electromagnetic Stirring. Metals.

[26] Shen J.L., Chen D.F., Xie X., Zhang L.L., Dong Z.H., Long M.J., Ruan X.B. (2013). Influences of SEN structures on flow characters, temperature field and shell distribution in 420 mm continuous casting mould. Ironmak. Steelmak..

[27] Chen W., Zhang Y.Z., Zhang C.J., Zhu L.G., Lu W.G., Wang B.X., Ma J.H. (2009). Thermo-mechanical simulation and parameters optimization for beam blank continuous casting. Mater. Sci. Eng. A.

[28] Li L., Wang X., Deng X., Wang X., Qin Y., Ji C. (2014). Application of High Speed Continuous Casting on Low Carbon Conventional Slab in SGJT. Steel Res. Int..

[29] Zhang L., Yang S., Cai K., Li J., Wan X., Thomas B.G. (2007). Investigation of Fluid Flow and Steel Cleanliness in the Continuous Casting Strand. Metall. Mater. Trans. B.

[30] Yoon J.-K. (2008). Applications of numerical simulation to continuous casting technology. ISIJ Int..

[31] Najjar F.M., Thomas B.G., Hershey D.E. (1995). Numerical study of steady turbulent flow through bifurcated nozzles in continuous casting. Metall. Mater. Trans. B.

[32] Zhao Y., Chen D.F., Long M.J., Shen J.L., Qin R.S. (2014). Two-dimensional heat transfer model for secondary cooling of continuously cast beam blanks. Ironmak. Steelmak..

[33] Xu H.L., Wen G.H., Sun W., Wang K.Z., Yan B. (2010). Analysis of Thermal Behavior for Beam Blank Continuous Casting Mold. J. Iron Steel Res. Int..

[34] Xu P., Yang C., Peng Y., Yao S., Zhang D., Li B. (2016). Crash performance and multi-objective optimization of a gradual energy-absorbing structure for subway vehicles. Int. J. Mech. Sci..

[35] Fu J., Liu Q., Liufu K., Deng Y., Fang J., Li Q. (2019). Design of bionic-bamboo thin-walled structures for energy absorption. Thin-Walled Struct..

[36] Song X., Sun G., Li G., Gao W., Li Q. (2012). Crashworthiness optimization of foam-filled tapered thin-walled structure using multiple surrogate models. Struct. Multidiscip. Optim..

[37] Zhu G., Wang Z., Cheng A., Li G. (2016). Design optimisation of composite bumper beam with variable cross-sections for automotive vehicle. Int. J. Crashworthiness.

[38] Angelis D., Sofos F., Karakasidis T.E. (2023). Artificial Intelligence in Physical Sciences: Symbolic Regression Trends and Perspectives. Arch. Comput. Methods Eng..

